# In Vitro and in Vivo Efficacy of Different Ointment Formulations Containing *Centaurium erythraea Rafn.* Aerial Extract

**DOI:** 10.3390/ph18111681

**Published:** 2025-11-06

**Authors:** Anett Jolán Karetka, Boglárka Papp, István Lekli, Ana-Maria Vlase, Annamária Pallag, Laura Grațiela Vicaș, Antonia-Maria Lestyán, Liza Józsa, Dóra Kósa, Ágota Pető, Zoltán Ujhelyi, Fruzsina Nacsa, Ildikó Bácskay, Pálma Fehér, Tünde Jurca

**Affiliations:** 1 Department of Pharmacy, Faculty of Medicine and Pharmacy, University of Oradea, 410068 Oradea, Romania; balasko.anettjolan@student.uoradea.ro (A.J.K.); apallag@uoradea.ro (A.P.); lvicas@uoradea.ro (L.G.V.); tjurca@uoradea.ro (T.J.); 2 Doctoral School of Biomedical Sciences, Faculty of Medicine and Pharmacy, University of Oradea, 410073 Oradea, Romania; antonia.lestyan@uoradea.ro; 3 Department of Pharmaceutical Technology, Faculty of Pharmacy, University of Debrecen, Rex Ferenc u.1., 4002 Debrecen, Hungary; papp.boglarka@pharm.unideb.hu (B.P.); jozsa.liza@pharm.unideb.hu (L.J.); bacskay.ildiko@pharm.unideb.hu (I.B.); 4 Doctoral School of Pharmaceutical Sciences, University of Debrecen, Nagyerdei krt.98., 4032 Debrecen, Hungary; ujhelyi.zoltan@pharm.unideb.hu; 5 Department of Pharmacodynamics, Faculty of Pharmacy, University of Debrecen, Rex Ferenc u.1., 4002 Debrecen, Hungary; lekli.istvan@pharm.unideb.hu; 6 Department of Pharmaceutical Botany, Faculty of Pharmacy, Iuliu Hatieganu University of Medicine and Pharmacy, 8 Victor Babes Street, 400012 Cluj-Napoca, Romania; gheldiu.ana@umfcluj.ro; 7 Department of Preclinical Disciplines, Faculty of Medicine and Pharmacy, University of Oradea, 410073 Oradea, Romania; 8 Department of Pharmaceutical Industry and Pharmaceutical Technology, University of Debrecen, Rex Ferenc u.1, 4002 Debrecen, Hungary; 9 MEDITOP Pharmaceutical Ltd., Pilisborosjenő Ady Endre u. 1, 2097 Pilisborosjenő, Hungary; fruzsina.nacsa@meditop.hu; 10 Pharmaceutical Ecosystem Center, University of Debrecen, Rex Ferenc u.1, 4002 Debrecen, Hungary

**Keywords:** *Centaurium erythraea*, DPPH, TPC, FRAP, in vivo anti-inflammatory effect, in vitro viability, Transcutol^®^ P, Capryol^®^ 90, ointment formulation

## Abstract

**Background:** *Centaurium erythraea Rafn*. (*C. erythraea*) is a medicinal plant traditionally used in European folk medicine for the treatment of wounds, skin inflammations, and other dermatological conditions, in addition to its well-documented systemic antioxidant and anti-inflammatory effects. However, its topical applications remain insufficiently investigated, particularly using plant material collected from Romania. The purpose of this study was to prepare different ointment formulations containing *C. erythraea* Rafn. extract obtained from the aerial parts of the plant, using various excipients, and to evaluate their in vitro and in vivo efficacy. **Methods:** The phytochemical profile of *C. erythraea* extract was characterized using liquid chromatography–tandem mass spectrometry (LC–MS/MS). The lyophilized extract was pre-dissolved in different solubilizing agents—Transcutol^®^ P (diethylene glycol monoethyl ether), Capryol^®^ 90 (propylene glycol monocaprylate), or a combination of both—and then incorporated into five ointment formulations. Texture analysis and an in vitro membrane diffusion study were performed. The antioxidant capacity of the formulations was assessed by 2,2-diphenyl-1-picrylhydrazyl (DPPH) radical scavenging, ferric reducing antioxidant power (FRAP), and total phenolic content (TPC) assays. Anti-inflammatory activity was evaluated in vitro using tumor necrosis factor-alpha (TNF-α)-induced interleukin-1 beta (IL-1β) production in human keratinocyte (HaCaT) cells, and in vivo using a carrageenan-induced rat paw edema model. **Results:** LC–MS/MS identified 18 polyphenolic compounds, with hyperoside (3.78 ± 0.05 µg/mL), protocatechuic acid (1.13 ± 0.06 µg/mL), chlorogenic acid (1.07 ± 0.06 µg/mL), and quercetin (0.53 ± 0.03 µg/mL) as the principal constituents. The formulation containing both Transcutol^®^ P and Capryol^®^ 90 exhibited the most pronounced antioxidant activity (65% DPPH inhibition; 69.71 ± 0.83 mg gallic acid equivalent/mL) and significantly reduced IL-1β levels by 45.7% compared to the inflamed control. In vivo, this formulation showed comparable anti-edematous effects to a methylprednisolone ointment. Furthermore, it demonstrated the highest skin permeation efficiency, with a quercetin diffusion coefficient of 35.12 × 10^−5^ cm^2^/min. **Conclusions:** These findings highlight the therapeutic potential of *C. erythraea* extract from aerial parts in topical formulations and underscore the enhancing role of Transcutol^®^ P and Capryol^®^ 90 in improving both the pharmacodynamic and pharmacokinetic properties of bioactive compounds.

## 1. Introduction

Herbs hold significant importance in human health due to their wide range of therapeutic effects [1]. Over the past thirty years, the global use of herbal medicines and supplements has risen dramatically, with more than 80% of people now depending on them for their primary healthcare needs [2]. As the demand for alternative therapies grows, it is increasingly essential to critically assess their safety and efficacy. Comprehensive scientific investigation is crucial to ensuring that these compounds offer therapeutic benefits while minimizing potential risks, facilitating their informed and responsible integration into healthcare practices [3]. *Centaurium erythraea* Rafn. (*C. erythraea*), the plant with a thousand medicinal properties according to Hungarian folk medicine, is an important healing plant from Gentianaceae family, which is used traditionally in the folk medicine as a digestive, stomachic, tonic, depurative, sedative, and antipyretic herb [4,5]. *C. erythraea* is also one of the potential drugs to treat asthma, eczema, jaundice, fever, intestinal parasites, rheumatism, hypertension, gastrointestinal muscle spasms, and edema and to stimulate the activity of the liver and gallbladder [6,7]. *C. erythraea* demonstrated significant antidiabetic properties by enhancing insulin sensitivity and lowering blood glucose levels, making it a promising natural treatment for diabetes [8,9]. The aerial parts of *C. erythraea* have also shown anti-inflammatory and antioxidant effects attributed to xantones and secoiridoid glycosides, namely gentiopicroside from the plant [10,11,12,13].

Antioxidants in skin formulations help protect against oxidative stress by neutralizing reactive oxygen species. This reduces free radical–induced cellular damage, supporting skin integrity and delaying visible signs of aging [14,15]. *C. erythraea* exhibits potent antioxidant activity attributable to its high concentration of polyphenolic compounds. These bioactive constituents contribute to the reduction in oxidative stress and modulation of inflammatory pathways, promoting cytoprotective effects within skin tissue [4,16].

Inflammation is a complex, well-regulated process the body uses to respond to injury or infection, characterized by pain, redness, heat, swelling, and sometimes loss of function [17,18]. This response includes recognition of danger signals, activation of pathways, and recruitment of immune cells [18,19]. While generally beneficial for tissue repair, persistent or dysregulated inflammation can become chronic and harmful, leading to permanent tissue damage [20]. Chronic inflammation is now recognized as a main factor in many major disorders, including pulmonary, metabolic and neurological diseases [21,22,23]. Topical inflammatory disorders including dermatitis and psoriasis are common examples where local tissue inflammation disrupts skin integrity, leading to discomfort and increasing health burden [24,25]. Although steroidal and non-steroidal anti-inflammatory drugs (NSAIDs) are effective, they often cause serious side effects and are not universally accessible [25,26]. In this context, medicinal herbs have appeared as promising alternatives, offering anti-inflammatory benefits with fewer adverse effects, greater affordability, and widespread availability [27]. Numerous studies have demonstrated the efficacy of plant-derived phytoconstituents, for instance, flavonoids, alkaloids, and phenolic compounds in modulating inflammatory pathways [28,29,30]. Therefore, exploring plant-based therapies is crucial for advancing safer and more effective management of inflammatory disorders [31].

Transcutol^®^ P (TP) and Capryol^®^ 90 (CP90) are widely used excipients in topical drug delivery systems, known for their ability to enhance the permeation of active pharmaceutical ingredients (APIs) through the skin [32]. TP is a diethylene glycol monoethyl ether with the chemical formula C_6_H_14_O_3_. This clear, low-viscosity liquid acts as a powerful solubilizer, significantly increasing the solubility of both hydrophilic and lipophilic drugs. Its polar nature allows it to disrupt the lipid structure of the stratum corneum, enhancing drug diffusion and facilitating deeper penetration into the skin layers [32,33].

CP90, on the other hand, is a propylene glycol monocaprylate, primarily composed of caprylic acid esters. With a chemical formula of C_10_H_20_O_3_*C*_10_*H*_20_*O*_3_, it serves as a nonionic surfactant that is a solubilizing agent and also enhances skin penetration by modifying the lipid composition of the stratum corneum. Its lipophilic characteristics improve drug solubility and facilitate the transport of APIs across the skin barrier [34].

Together, these excipients demonstrate a synergistic effect in topical formulations, where TP increases drug solubility while CP90 enhances permeation, leading to improved bioavailability and therapeutic efficacy of topical medications [35].

Ointments are semi-solid formulations that play a significant role in topical drug delivery systems, primarily due to their capacity to provide localized treatment while minimizing systemic absorption. These formulations are typically composed of a base that may be oleaginous, absorption-type, or emulsion-based, which facilitates the effective solubilization and penetration of active pharmaceutical ingredients (APIs) through the skin barrier. A notable advantage of ointments lies in their ability to deliver medications directly to specific areas of the skin. This targeted application is particularly advantageous for the treatment of localized conditions, such as dermatological disorders or localized pain. The oily composition of ointments enhances the solubilization of lipophilic drugs, thereby improving their absorption through the stratum corneum, which constitutes a significant barrier to drug entry into the systemic circulation. Furthermore, ointments form a protective layer on the skin that aids in moisture retention, making them particularly suitable for managing dry or irritated skin conditions [3,36,37].

Another important aspect regarding the examination of our selected medicinal plant is that there is no study of the bioactive components of *C. erythraea* collected in our country. A detailed review published in 2021 collected studies from all countries concerning the phytochemical studies of *C. erythraea*, and the data published from Romania is very incomplete [10,38]. Among other things, the purpose of our research is to improve the existing bibliography because the number of sites where this plant occurs is very limited. Even though there are few studies on the medicinal plant, its medicinal use is widespread in several countries; in Poland, for example, about 10 tons of the raw materials of *C. erythraea* are collected each year [39]. The convergence of ethnomedical documentation and contemporary pharmacological evidence underscores the therapeutic relevance of this species within traditional European medicine. Phytochemical analyses have revealed a complex profile dominated by xanthonoids, flavonoids, and phenolic acids, compounds known to mediate a broad spectrum of bioactivities. Such findings substantiate its empirical use as a bitter tonic, diuretic, and anti-inflammatory agent, providing a mechanistic basis for its antioxidant, hepatoprotective, and gastroprotective effects [4,10,40].

The main objectives of the present study were to formulate ointments containing *C. erythrea* extract with different excipients that have solubilizing and penetration enhancer effects in order to achieve a good diffusion profile with antioxidant and anti-inflammatory effects.

## 2. Results

### 2.1. Phytochemical Profile

The phytochemical composition of *C. erythraea* extract was analyzed using LC-MS/MS, enabling the identification and quantification of major bioactive compounds. Table 1 provides a comprehensive summary of the identified and quantified compounds, obtained through both validated LC-MS/MS methods.

The LC-MS/MS analysis of *C. erythraea* extract revealed the presence of various phenolic acids and flavonoids, compounds known for their antioxidant, anti-inflammatory, and wound-healing properties. The phytochemical profile of *C. erythraea* reveals that several polyphenolic constituents are notably abundant in the lyophilized extract. Hyperoside stands out as the most prevalent compound at 3.78 ± 0.05 µg/mL, followed by protocatechuic acid (1.13 ± 0.06 µg/mL), chlorogenic acid (1.07 ± 0.06 µg/mL), and 4-O-caffeoylquinic acid (0.97 ± 0.06 µg/mL). Isoquercitrin is also present in a considerable amount (0.66 ± 0.06 µg/mL). The prominence of these specific polyphenols suggests they are likely the primary contributors to the extract’s antioxidant and anti-inflammatory activities, underpinning the therapeutic effects observed in the study. This quantitative distribution highlights the rich polyphenolic nature of the Romanian *C. erythraea* extract and provides a basis for understanding its pharmacological potential.

### 2.2. Determination of Particle Size, PDI and Zeta Potential of Pre-Dissolved C. erythraea Lyophilized Product

Measurable microparticles were detected, which remained within the microscale range even after 30 days. However, slight aggregation was observed in all formulations, with a more pronounced effect in the CP90-containing formulation. The zeta potential results support these findings, as lower values (e.g., around −6.45 mV) indicate moderate stability. Results are shown in Table 2. In general, a zeta potential exceeding ±30 mV indicates good colloidal stability, whereas values below ±10 mV suggest weak stability and a higher tendency for particle aggregation [41].

### 2.3. Results of Texture Analysis

**Figure 1:**
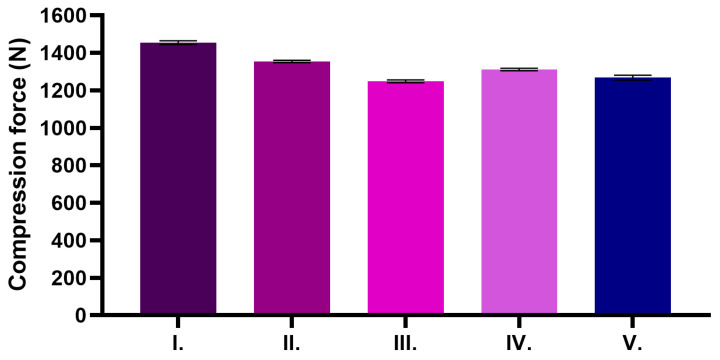
Texture analysis of ointments was performed at 25 °C, with measurements recorded as compression force. Results are expressed as mean ± SD, based on six replicates (*n* = 6).

The results of the texture analysis are shown in Figure 1. In the study, the formulations were compared with or without excipients in the formulations containing the active ingredient and without the active ingredient. The base cream without the active ingredient showed the highest resistance to the test probe, suggesting that it is the most resistant formulation. Ointments containing TP showed lower resistance; these ointments are probably softer.

### 2.4. Results of pH Measurement

The pH values of the ointment formulations remained within the physiological range of normal skin pH (approximately 4.5–6.5) throughout the 30-day period. No significant pH changes were observed, indicating that the formulations maintained chemical stability and skin compatibility over time. The initial and 30-day pH values are presented in Table 3.

### 2.5. Results of Rheological Properties of Ointments

**Figure 2:**
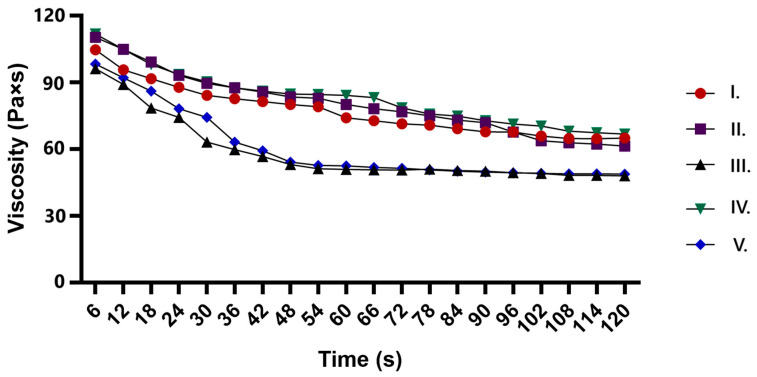
Viscosity profiles of different ointment formulations over time, showing shear-thinning behavior. Formulation II. contains only the API, while Formulations III, IV, and V include Transcutol P, Capryol 90, or both excipients, respectively.

The viscosity profiles of the ointments showed shear-thinning behavior, with all formulations decreasing in viscosity over time. The API-only formulation (II.) and the CP90-containing formulation (IV.) maintained higher viscosity, indicating a more structured network. In contrast, formulations with TP (III. and V.) exhibited a significant drop in viscosity, suggesting its role in reducing structural integrity. The combination of CP90 and TP (V.) resulted in the lowest viscosity, indicating a synergistic effect in breaking down the formulation structure. These results confirm that the ointments exhibit pseudoplastic behavior, and the choice of excipients significantly influences their rheological properties. All the results are shown in Figure 2.

### 2.6. Results of Synthetic Membrane Diffusion Model

Each composition contained 1.00 g of lyophilized *C. erythraea*, which is equivalent to 5.27 ± 2.6 µg quercetin.

**Figure 3:**
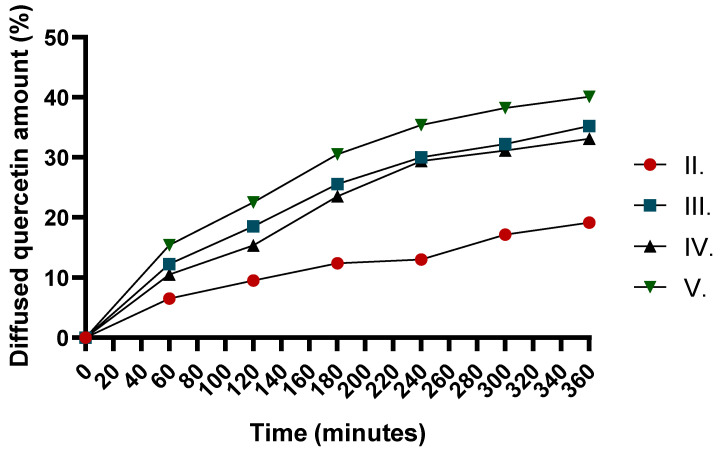
Diffusion profile of quercetin from different formulations over time.

The dissolution of quercetin from the formulations varied significantly over time, with formulation V. (Figure 3.) showing the highest diffusion rate. Compared to formulation II., which contained only the API, formulation III. and IV. exhibited enhanced dissolution due to the presence of TP and CP90, respectively (Table 4). TP, a solubilizer, may enhance drug solubility, while CP90, a lipophilic surfactant, could promote better penetration and diffusion. Therefore, the combined effect of these excipients in formulation V. leads to the highest dissolution, making it a promising formulation for improved drug delivery. The kinetic evaluation of the release profiles further supports these findings (Table 5).

### 2.7. Antioxidant Effect of C. erythraea-Containing Ointments

#### 2.7.1. DPPH

**Figure 4:**
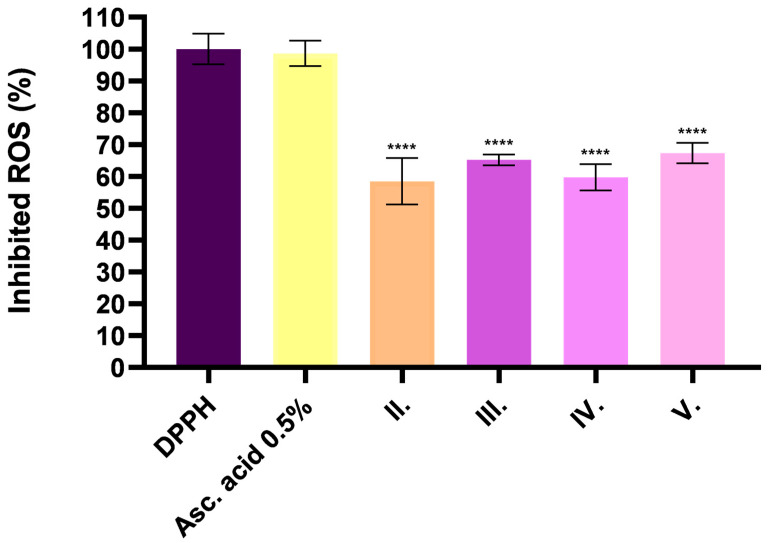
DPPH antioxidant activity study on *C. erythraea* formulations with different excipients. Ascorbic acid 0.5 *w*/*v*% was used as a positive control. Results are expressed as mean ± S.D. All data are the mean of at least 6 measurement points (*n* = 6). **** indicates a statistically significant difference (*p* < 0.0001) compared to the control group.

Based on the results of the DPPH antioxidant test, the antioxidant activity of ointments containing *C. erythraea* extract was observed to be heterogeneous between different formulations. Ointment containing the herbal extract alone (II.) showed 58% DPPH radical scavenging activity. For the ointment containing both TP and herbal extracts (III.), the antioxidant activity increased to 62%, while the formulation combining CP90 and herbal extract (IV.) showed 59% activity. The formulation containing TP, CP 90 and herb (V.) showed the highest radical scavenging activity (65%), which may indicate a synergistic effect of the ingredients. A 0.5% ascorbic acid solution was used as a positive control, as its strong antioxidant effect is well known and provided a reference for comparison. The radical scavenging properties of the formulations are shown in Figure 4.

#### 2.7.2. FRAP Assay

**Figure 5:**
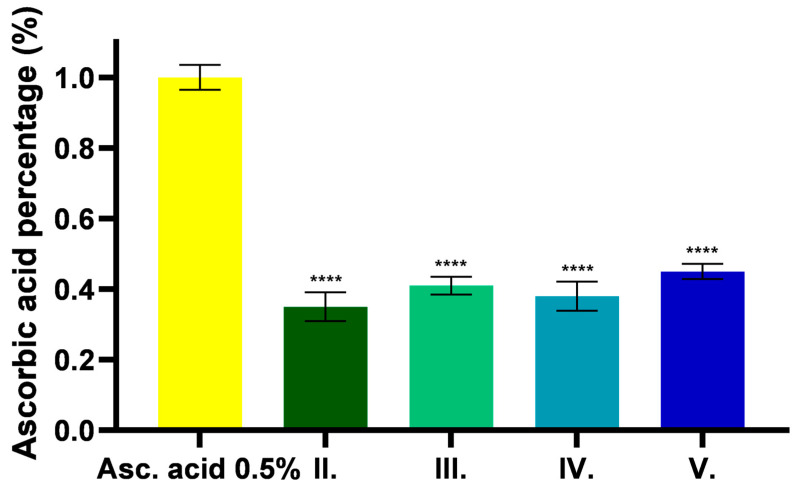
Results of an antioxidant test based on ferric reducing capacity for different formulations containing *C. erythraea*. Results are expressed as percentage of mean + SD, *n* = 6. **** indicates a statistically significant difference (*p* < 0.0001) compared to the control group.

The FRAP assay expresses the reducing ability of the tested samples in acidic environments. The results are presented in Figure 5, expressed as a percentage of ascorbic acid. The results show that *C. erythraea* has antioxidant property in acidic environment, which showed higher values in the presence of excipients. Higher capacity was measured for samples containing TP than for samples without excipients or containing only CP90.

#### 2.7.3. Determination of TPC (Total Phenolic Content)

The total phenolic content (TPC) of the samples was determined using the Folin–Ciocalteu method and expressed as milligrams of gallic acid equivalents per milliliter (mg GAE/mL). The TPC for formulation II. was found to be 59.07 ± 4.43 mg GAE/mL. Formulation III. had a TPC of 68.43 ± 1.84 mg GAE/mL, while formulation IV. measured 66.84 ± 0.57 mg GAE/mL. The highest TPC was observed in formulation V. at 69.71 ± 0.83 mg GAE/mL, indicating that this sample had the most significant phenolic content among those tested (Table 6).

### 2.8. MTT Cytotoxicity Assay

**Figure 6:**
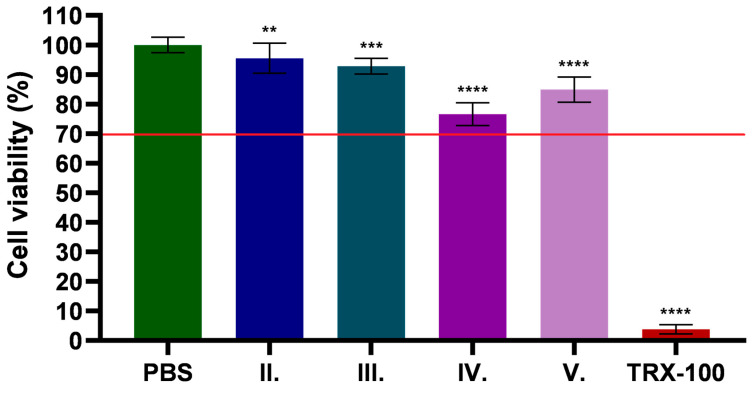
Cytotoxicity assay of *C. erythraea* extract formulations on human keratinocyte cells (HaCaT). PBS was used as a negative control and the results are expressed as a percentage of the mean ± SD (*n* = 6). The red line represents the 70% cell viability threshold. **, ***, and **** indicate statistically significant differences compared to the control group at *p* < 0.05, *p* < 0.001, and *p* < 0.0001, respectively.

The results of the MTT test are shown in Figure 6. Results are expressed as a percentage of the negative control. PBS was used as negative control and Triton-X 100 (TRX-100) (1.00 V/V%) as positive control. Based on the results, it can be shown that the API was well tolerated and safe for the cells in all cases, with viability values of all formulations (II.–V.) higher than 70%, which is in accordance with the recommendations of ISO 10993-5:2009. 

In the case of the CP90 containing ointment formulation, slightly lower viability values were measured, whereas higher cell viability was measured for the TP containing ointments.

### 2.9. In Vitro Anti-Inflammatory Effect of C. erythraea Extract

**Figure 7:**
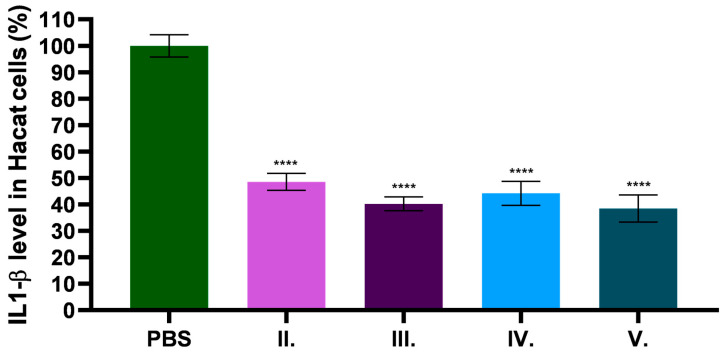
The percentage of IL-1β level in the HaCaT cell line. To compare the different formulations with PBS, a normal one-way ANOVA with Dunnett’s multiple comparison test was performed. **** indicate statistically significant differences at *p* < 0.0001, respectively. Results are expressed as mean ± SD (*n* = 6).

The anti-inflammatory effect of *C. erythraea*-containing formulations was tested by IL-1β sandwich ELISA on HaCaT cell line. The samples were previously dissolved in PBS. The negative control was the PBS to which the test substances were compared and expressed as a percentage. Figure 7 shows the results of the anti-inflammatory effect on the HaCaT cell line expressed as a percentage of IL-1β levels. The results of the study show that *C. erythraea* has a significant anti-inflammatory effect. In the presence of excipients, the anti-inflammatory effect was enhanced in all three formulations (III.–V.). The formulation containing both excipients showed the highest anti-inflammatory effect.

### 2.10. Results of In Vivo Carrageenan-Induced Rat Paw Edema Model

**Figure 8:**
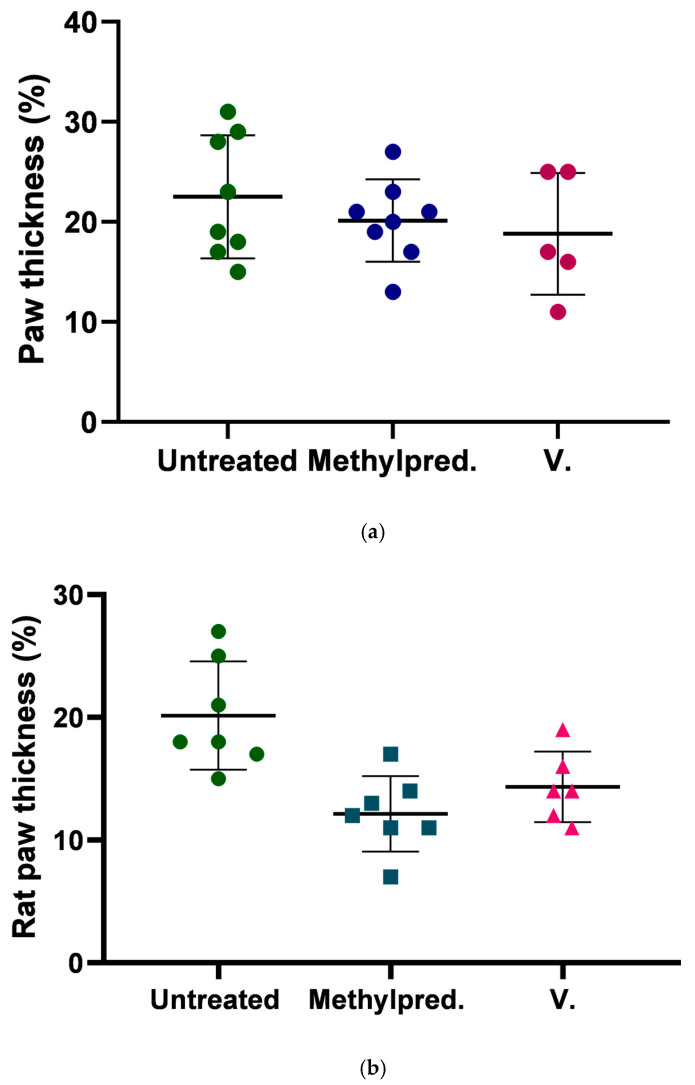
Carrageenan-induced rat paw edema study. The study was repeated three times using 3 animals. The 3 h results (**a**) and 24 h results (**b**) were compared to untreated control and positive control. Results are expressed as the SD ± mean.

The effect of our herb-containing ointment on edema was investigated in a subplantar carrageenan solution-induced paw edema study, in combination with untreated paws as untreated controls and with methylprednisolone ointment (1 mg/g) applied as a positive control. The results shown in Figure 8. and represent that the rat paw size increased significantly after 3 h in untreated controls, while the positive control and the herb ointment produced less edema. At 24 h, the swelling of the untreated control was reduced to a lower extent than that of the positive control or the medicinal herbal ointment. Comparing the results of the positive control with the results of the herbal ointment at the 3 h measurement, a slightly lower value was obtained for the percentage of swelling in the herbal ointment. In the 24 h results, a decrease in swelling was measured for the positive control compared to the ointment containing *C. erythraea*.

## 3. Discussion

This study aims to address the limited research on *C. erythraea*, particularly regarding its topical uses, despite its long history in traditional medicine. Although the plant has been extensively studied for its benefits related to digestion and metabolic health, there is a clear lack of scientific exploration on its bioactive components from our region, with no thorough studies conducted on specimens collected locally [10].

A literature search was conducted using Google Scholar with the search terms “*Centaurium erythraea* oral” (1710 articles) and “*Centaurium erythraea* topical” (504 articles). The results revealed that studies on oral (digestive) administration outnumber those focused on topical applications by a factor of about 3.4 times, emphasizing the stronger research focus on oral use. This highlights the need for further exploration of its potential in topical formulations.

Geographical location significantly influences a plant’s phytochemical content due to factors such as climate, soil type, and environmental conditions. Variations in temperature, sunlight, humidity, and soil nutrients across regions can alter the synthesis and accumulation of secondary metabolites, resulting in differences in phytochemical profiles [42].

*C. erythraea* is widely distributed in Romania and shows a preference for open and moderately dry environments. It is commonly found in meadows, at the edges of plain forests, and in hill areas [43]. The species prefers calcareous soils and dry grassy places, including sand dunes and chalky uplands, which are common in its Romanian habitats [43,44]. This adaptability to a variety of soils and moderately disturbed environments makes *C. erythraea* a characteristic component of Romania’s flora, thriving especially well in semi-natural grasslands and similar ecosystems [45].

A study from 2019 concluded that 70% ethanol is a more efficient solvent than water for extracting compounds from *C. erythraea*. Ethanolic extracts, such as tincture, resulted in higher phenol, flavonoid and anthocyanin contents than aqueous extracts, such as infusions or microwave extractions. The research highlights that solvent polarity plays an important role in increasing extraction yields, as 70% ethanol is better able to dissolve phenolic compounds. The results show that ethanol has a better extractability, resulting in higher concentrations of the targeted compounds [46].

The tincture has been lyophilized as these kinds of extracts are highly preferred in pharmaceutical applications because they enhance the stability and shelf-life of bioactive compounds, facilitating long-term storage and reducing the need for cold chain logistics [47]. As an example, a study with a lyophilized *Ficus capensis* extract, another high-polyphenol plant, has shown its ability to alleviate chemotherapy-induced liver damage in rats due to its high phenolic content, proving its therapeutic efficacy while preserving bioactive compounds during storage and treatment [48,49].

The HPLC analysis in combination with LC-MS/MS was crucial for the identification and quantification of the 18 polyphenolic compounds present in the *C. erythraea* extract. This sophisticated analytical method allowed the correct characterization of bioactive compounds using two validated approaches, providing a comprehensive evaluation of compounds such as flavonoids and polyphenolic acids. Of the main bioactive compounds identified, chlorogenic acid and its derivative, 4-*O*-caffeoylquinic acid, exhibit potent antioxidant and anti-inflammatory properties. These compounds play an important role in reducing oxidative stress, which is a key factor in delayed wound healing and chronic inflammation. Studies have shown that chlorogenic acid can modulate inflammatory pathways, reducing the excessive production of reactive oxygen species (ROS) and thereby protecting skin cells from oxidative damage [50]. This mechanism supports the potential topical use of *C. erythraea* extract in wound care formulations.

In addition, caffeic and ferulic acids contribute significantly to skin regeneration and collagen synthesis, which are fundamental processes in wound healing. Caffeic acid has been shown to enhance fibroblast proliferation, while ferulic acid strengthens the skin barrier, making it more resistant to external injury [51,52,53]. Their presence in *C. erythraea* extract suggests that it could be a valuable phytotherapeutic agent for promoting dermal repair.

Another important compound found in the extract is protocatechuic acid. This phenolic acid is widely recognized for its anti-inflammatory and antimicrobial properties, which are particularly beneficial in reducing bacterial infections and controlling inflammation in damaged skin. Its dual action—reducing inflammation while preventing microbial colonization—makes it a promising candidate for topical formulations aimed at accelerating the healing process of infected wounds. Furthermore, protocatechuic acid has been shown to stimulate the synthesis of type I collagen in human dermal fibroblasts while simultaneously inhibiting MMP-1 secretion in UVA-irradiated fibroblasts, highlighting its potential anti-wrinkle and anti-aging properties [54].

Moreover, gallic acid, a well-known polyphenol with potent antimicrobial, antiviral, anti-cancer, and anti-inflammatory properties, has traditionally been associated with numerous health benefits. Beyond these properties, gallic acid has also been shown to directly upregulate the expression of antioxidant genes, reinforcing its role in cellular protection against oxidative stress [55]. Furthermore, gallic acid can enhance keratinocyte and fibroblast migration, essential processes in wound closure and regeneration. This effect is further supported by the ability of gallic acid to activate key wound-healing signaling pathways, including focal adhesion kinases (FAK), c-Jun N-terminal kinases (JNK), and extracellular signal-regulated kinases (Erk), all of which are critical for tissue remodeling and repair [56]. Therefore, *C. erythraea* extract, due to its gallic acid content, may have benefits in managing inflammation and wound healing.

In addition to the phenolic acids *C. erythraea* extract contains quercetin and flavonolic glycosides, particularly hyperoside, quercetin and quercitrin, compounds that are renowned for their potent antioxidant, anti-inflammatory, antimicrobial, photoprotective, anti-age, and wound-healing effects [57,58,59,60]. These compounds play a crucial role in modulating inflammatory pathways, reducing proinflammatory cytokines and enhancing epithelial regeneration. Their presence in *C. erythraea* supports its traditional use in wound treatment, as flavonols are known to stimulate proliferation and migration of keratinocytes, key factors in tissue repair. Furthermore, isoquercitrin, another flavonolic glycoside present in the extract, has been reported to specifically inhibit proinflammatory cytokines, supporting its potential role in reducing inflammation and topical wound treatments [61,62]. Its ability to modulate immune responses and limit excessive inflammation makes it particularly useful in the treatment of inflammatory conditions such as chronic wounds and rashes.

*C. erythraea* extract shows strong antioxidant and anti-inflammatory properties, principally through chlorogenic acid and its derivative 4-O-caffeoylquinic acid, which help to reduce oxidative stress and protect skin cells [63]. In addition, gallic acid upregulates antioxidant genes, while isoquercitrin specifically inhibits proinflammatory cytokines, supporting its therapeutic potential in inflammatory disorders [64,65].

In our analysis of *C. erythraea* ethanol extract, hyperoside was the most abundant polyphenol at 3.78 µg/mL. This contrasts with most studies where swertiamarin, a secoiridoid, predominates. For example, Aberham et al. found swertiamarin levels up to 113 mg/g in dried plant material, far exceeding hyperoside and flavonoids [12]. Similarly, Stefkov et al. reported swertiamarin as the main compound in methanolic extracts [66]. The choice of solvent is crucial for targeting specific therapeutic effects. Methanol extracts yield higher secoiridoid levels like swertiamarin, which are linked to digestive and hypoglycemic benefits, making them suitable for oral use. In contrast, ethanol (especially 70% hydroalcoholic) extracts are richer in polyphenols and flavonoids, which provide strong antioxidant and anti-inflammatory effects [10]. Therefore, ethanol extracts are ideal for topical applications aimed at reducing inflammation and oxidative stress.

To assess the physical stability of the formulations, the study monitored the most important properties for 30 days. The microparticles remained within the micron size range in all samples; however, some degree of aggregation was observed, specifically in the formulations containing CP90. Other studies have shown that systems containing CP90 may exhibit specific behavior, especially in terms of stability. These investigations have shown that, even though mixtures containing CP90 may appear homogeneous at the macroscopic level, considerable heterogeneities may arise at the microscopic structure level. These minor phase differences can lead to phase separation over time, especially during long-term storage. This suggests that the stability problems of CP90 are not simply a function of physical miscibility, but that underlying complex component interactions also play a role in the behavior of the system [34,67].

Skin pH plays a major role in determining the effectiveness of topical products. The acidic pH of the skin surface (between 4.1 and 5.8) regulates a number of important processes that influence the efficacy of topical formulations [68]. This acidic environment is necessary for the optimal activity of important skin enzymes, with β-glucocerebrosidase functioning best at pH 5.5 and acid sphingomyelinase at pH 5 [69,70].

Products with a pH outside this range can disrupt the barrier function of the skin and reduce the effectiveness of the product. The pH of the products also affects the penetration of the active ingredients and their ability to interact with skin structures [71]. The pH of the topical products also affects the penetration of the active ingredients [72]. This understanding has led to more efficient development of formulations to support skin health. In our study, pH values remained relatively stable throughout the study and consistently remained within the physiological range suitable for application to the skin.

In our approach to improve the performance of ointment formulations, TP and CP90 were selected strategically based on their well-demonstrated ability to promote drug solubilization and transdermal absorption [73].

TP was chosen mainly for its multifunctional role as both a strong solvent and penetration enhancer [74]. This excipient considerably improves the solubility of the active ingredient within the formulation and facilitates diffusion across the skin barrier by modifying the solubility of the active ingredient in the stratum corneum [75]. This is obtained through mechanisms such as solvent resistance, where drug molecules are carried alongside the TP penetrates the skin. Its biocompatibility, biodegradability, and miscibility with polar and non-polar materials make it a multifunctional component of topical systems [76]. Significantly, TP enhances permeation without disrupting the skin barrier or causing irritation, underscoring its safety and efficacy for dermatological use [77].

CP90 was chosen for its excellent solubilizing ability of lipophilic drugs, with a high solubility value of approximately 305 mg/g. Used in combination with medium-chain triglycerides in an optimized 2:1 ratio, CP90 contributes to achieving an ideal hydrophilic-lipophilic balance (HLB), thus promoting higher drug loading and improving percutaneous absorption. Additionally, its incorporation into nanoemulsion systems improves drug deposition in deeper skin layers by more than four times compared to conventional formulations. The moderate HLB value (HLB = 5) also enhances the stability of the formulation and efficient delivery of the active ingredients [78].

Together, TP and CP90 offer additional benefits that are in line with modern strategies for optimizing skin delivery, and are therefore integral to the improved performance of our ointment formulation.

Texture analysis studies have shown that TP has a major impact on the physical properties and resistance of formulations. Above a concentration of 25 wt.% TP, significant disturbances in the microstructure of the creams and reduced resistance were observed [33]. This is confirmed by our results where texture analysis showed that the base cream without active ingredients showed the highest resistance when tested with the probe, making it the most resistant formulation. In contrast, the TP containing ointments showed lower resistance values, suggesting that they were softer in consistency.

Rheological behavior plays a critical role in topical formulations by providing early indicators of potential product failure, as it controls the structural stability, consistency and resistance to external forces of the topical formulation, while ensuring optimal spreadability, storage stability and overall product performance by monitoring viscoelastic properties and structural changes over time [79].

An interesting discovery in our study was the synergistic effect that we observed when combining CP and TP in ointment formulations. Specifically, this combination resulted in the lowest viscosity of all the formulations tested, suggesting a significant effect on the structural integrity of the ointment. This result highlights that interactions of excipients can dramatically alter the rheological properties of topical formulations, offering new opportunities for optimization of drug delivery systems. The synergistic viscosity reduction achieved by CP and TP highlights the importance of careful selection and combination of excipients to obtain the preferred formulation characteristics.

This synergistic effect was also demonstrated in diffusion analyses, where the combination of CP90 and TP resulted in the highest diffusion rate. This dual benefit—reduced viscosity and enhanced drug dissolution—makes this formulation extremely promising for improved drug delivery.

The existing literature has shown good antioxidant activity by various extraction methods, with hydroalcoholic extracts demonstrating especially high efficiency, as evidenced by TEAC values of 48.6 ± 0.4 μmol TE/g dry weight in DPPH assays [80]. In addition, the aerial parts of *C. erythraea* presented promising results in FRAP (Ferric Reducing Antioxidant Power) assays, further highlighting its robust antioxidant capacity [81]. Moreover, the bioactive compounds in the plant act synergistically, combining hydrophilic and lipophilic antioxidants to provide comprehensive protection against oxidative stress by inhibiting lipid peroxidation and reducing oxidative damage [16]. In our study we also found that antioxidant analysis methods (DPPH, FRAP and TPC) showed significant correlations in the results and consistent patterns in the different formulations. Most importantly, all three methods demonstrated increased antioxidant activity in formulations containing excipients, especially when both TP and CP90 were present. These results were not obtained by coincidence; other studies also point to the fact that TP and CP90 are employed in formulations to enhance the solubility and stability of bioactive compounds, particularly phenolics. By stabilizing these compounds and preventing degradation or oxidation, these solubilizers can lead to increased detected levels of total phenolic content (TPC) [82,83].

Keratinocytes, which constitute about 90% of the epidermis—the most external layer of the skin—are the main cellular component of this protective barrier [84]. As the body’s first line of defense, keratinocytes play a key role in the skin by acting as the primary barrier to the epidermis and actively participate in wound healing through proliferation and migration, restoring skin integrity and maintaining homeostasis, while also providing protection against oxidative stress and helping to reduce inflammation during tissue repair [85]. Out of the various human keratinocyte models, the HaCaT cell line is notable for its spontaneous immortalization and epithelial origin [86]. These cells are commonly used in in vitro studies because they accurately represent the primary cell type of the epidermis, making them particularly valuable for evaluating topical formulations and their effects on skin biology. In our study we also used HaCaT cell line, and the MTT cytotoxicity results showed that the drug was well tolerated and safe in all formulations. The formulations containing CP90 showed slightly reduced cell viability, while the formulations containing TP showed increased viability.

This is shown in another MTT cytotoxicity study, in which formulations containing CP90 showed moderate toxicity, resulting in slightly reduced cell viability compared to other formulations. On the other hand, formulations containing TP presented low toxicity and increased cell viability, indicating an excellent safety profile [87]. Overall, the results indicate that although all formulations are generally well tolerated, those containing TP are particularly suitable for applications where minimal impact on cell viability is crucial.

Recent research highlights that IL-1β plays a fundamental role in driving skin inflammation in atopic dermatitis. Studies using filaggrin-mutant mice demonstrate that IL-1β production is significantly elevated in inflamed skin and that blocking IL-1β signaling reduces inflammation, suggesting its critical importance in the pathogenesis of atopic dermatitis [88]. Our results showed that *C. erythraea* produced a significant anti-inflammatory effect. All preparations containing excipients showed an increased anti-inflammatory effect compared to the base preparation. Especially the preparation containing both excipients showed the strongest anti-inflammatory effect. These results suggest that the combination of TP and CP90 not only improved the physical properties of the preparation but also enhanced the therapeutic anti-inflammatory potential. This can be explained by the combination of TP and CP90 improving drug delivery, as TP acts as an effective absorption promoter and solubilizer, being able to mix with both polar and non-polar solvents, while CP90 acts as a surfactant to improve the physicochemical stability and miscibility of the formulation [32].

In vivo testing is essential in scientific research as it bridges the gap between in vitro results and real biological conditions, and offers fundamental insights into safety, efficacy and biological interactions that cannot be fully replicated in the laboratory.

In terms of promising in vitro anti-inflammatory results, carrageenan is given to rats in most of the in vivo experiments because it creates a well-researched and reproducible model of inflammation. When injected into the hind paws of rats, it quickly induces cardinal signs of inflammation, including edema, hyperalgesia and erythema in response to inflammatory substances such as bradykinin, histamine and prostaglandins [89]. The results showed that after 3 h, untreated controls had significant paw swelling, while both the herbal ointment and the positive control (methylprednisolone) reduced edema. After 24 h, the herbal ointment showed similar anti-inflammatory effects as the positive control. The anti-inflammatory effect is similar; however, the side effect list of methylprednisolone aceponate (MPA) should not be underestimated. These include skin dryness, mild erythema, burning sensation, scaling and rash [90]. Less frequent reactions include skin dryness, fissures, pain, vesicles, pustules, erosion, and redness [91]. Importantly, MPA has a low risk of skin atrophy compared to other potent corticosteroids and minimal systemic effects have been reported but nevertheless more significant side effects than *C. erythraea*-based formulations.

## 4. Materials and Methods

3-(4,5-Dimethylthiazol-2-yl)-2,5-diphenyltetrazolium bromide (MTT powder), Dulbecco’s Modified Eagle’s Medium (DMEM), phosphate-buffered saline (PBS), TrypLE™ Express Enzyme, heat-inactivated fetal bovine serum (FBS), L-glutamine, non-essential amino-acid solution, and penicillin–streptomycin were purchased from Sigma Aldrich (Sigma Aldrich, St. Louis, MO, USA). Culturing flasks and 96-well plates were purchased from Thermo-Fisher (Darmstadt, Germany, CAS number: 156499). Cetostearyl alcohol, white petroleum, beeswax, polysorbate 60 and conserving solution were obtained from Hungaropharma Ltd. (Budapest, Hungary). HaCaT cells were supplied from Cell Lines Service (CLS, Heidelberg, Germany). Transcutol^®^ P and Capryol^®^ 90 were a kind gift from Gattefossé (Lyon, France). FRAP assay (P. No.:MAK509), 2,2-diphenyl-1-picrylhydrazyl (DPPH), gallic acid monohydrate (CAS Number: 5995-86-8), Folin and Ciocalteu′s phenol reagent, sodium carbonate decahydrate (CAS Number: 6132-02-1), human IL-1β ELISA Kits were purchased from Sigma-Aldrich (Budapest, Hungary).

### 4.1. Preparation of C. erythraea Lyophilized Product

The plant material-herba was obtained in dry form from a national herbal tea producer (SC STEF MAR SRL, Valcea, Romania). The plants were collected from Valcea county (Romania). The aerial parts (herba, stems, leaves, and flowers) of *C. erythraea* were selected for the study because, according to the literature, these parts are known to contain the highest concentrations of bioactive compounds responsible for the therapeutic properties of the plant and are commonly used in in vitro and in vivo studies [10].

The tincture (1:10) was obtained from 10 g of plant material and 100 mL 70% ethanol, by maceration for 7 days at RT. The solvent was evaporated with R-300 rotary evaporator (Heidolph Instruments, Schwabach, Germany) at a temperature 50 °C under vacuum at 200 mBarr and then lyophilized.

*C. erythraea* was used and analyzed by LC-MS/MS to identify and quantify the main bioactive compounds of the plant. In the present study, lyophilized plant material was used for all further analyses.

### 4.2. Phytochemical Analysis Using LC-MS/MS

The phytochemical profile of *C. erythraea* extract was characterized using liquid chromatography-tandem mass spectrometry (LC-MS/MS), applying two validated analytical methods optimized for the identification and quantification of polyphenolic compounds [92,93,94,95]. The analyses were carried out using an Agilent Technologies 1100 HPLC Series system (Agilent, Santa Clara, CA, USA), which included an autosampler, column thermostat, binary gradient pump, degasser, and UV detector [92,93]. This system was coupled with an Agilent Ion Trap 1100 SL mass spectrometer (LC/MSD Ion Trap VL) [94,95,96].

The first LC-MS method was developed for the detection of 28 polyphenols, ensuring a comprehensive assessment of the major bioactive compounds present in the extract. Chromatographic separation was performed using a Zorbax SB-C18 reverse-phase analytical column (100 mm × 3.0 mm i.d., 3.5 μm particle size, Agilent Technologies, Santa Clara, CA, USA), with a mobile phase consisting of methanol and 0.1% acetic acid (*V*/*V*) in binary gradient mode. The gradient started at 5% methanol, progressively increasing to 42% over 35 min, followed by an isocratic phase at 42% methanol for 3 min, and a re-equilibration step at 5% methanol over 7 min. The column was maintained at 48 °C, with a flow rate of 1 mL/min and an injection volume of 5 μL. UV detection was performed at 330 nm for polyphenolic acids (0–17 min) and 370 nm for flavonoids and aglycones (17–38 min). The mass spectrometric analysis was conducted in negative electrospray ionization (ESI) mode, with a capillary voltage of +3000 V, a nebulizer pressure of 60 psi (nitrogen), and a gas flow rate of 12 L/min at 360 °C [92,93,94].

A second LC-MS method was used to expand the profiling of eight additional polyphenolic compounds, including epicatechin, catechin, syringic acid, gallic acid, protocatechuic acid, vanillic acid, epigallocatechin, and epigallocatechin gallate. The same chromatographic column and equipment were used, but with a modified gradient program: the methanol concentration started at 3%, increased to 8% at 3 min, reached 20% at 8.5 min, and was maintained until 10 min, before returning to 3% methanol. MS detection was performed under the same ESI conditions as the first method.

Compound identification was achieved by comparing MS spectra and chromatographic traces with library standards, while quantification was performed using UV detection, based on calibration curves of corresponding analytical standards [95].

Data acquisition and processing were carried out using DataAnalysis (v5.3) and ChemStation (vB01.03) software (Agilent, Santa Clara, CA, USA) [25,26,27,28]. Results were expressed as micrograms of bioactive compound per milliliter of extract.

### 4.3. Measurement of Particle Size, PDI and Zeta Size of Pre-Dissolved C. erythraea Lyophilized Product

In order to solubilize and reduce the particle size of *C. erythraea* lyophilized product it was pre-dissolved in TP (1.), in CP90 (2.) and in the mixture of TP-CP90 (3.) as Table 7 shows. The particle size of these formulations was determined by dynamic light scattering using a Zetasizer Nano S (Malvern Instruments Ltd., Malvern, UK). Dilutions of 100-fold in distilled water were used to dilute test samples. To evaluate the stability of the samples, the particle size was measured on the day of production (day 0) and on day 30 after production (day 30). The results were characterized by the polydispersity index (PDI) [97].

The electrostatic potential, or zeta potential, of the bilayer surrounding the droplets was measured using Zetasizer Nano S instrument (Malvern Instruments Ltd., Malvern, UK). This analysis provided important information on the colloidal stability of self-emulsifying systems, as the value of the zeta potential determines the degree of electrostatic repulsion between particles. Samples were prepared freshly with distilled water at 100-fold dilutions to ensure adequate measurement conditions and to minimize variations due to concentration. For more accurate results, all measurements were repeated three times, and the average zeta potential values were analyzed.

### 4.4. Formulation of Ointments Containing C. erythraea Lyophilized Product

To prepare the formulations, first an *o*/*w* emulsion ointment base was formulated. The oily phase consisting of a mixture of white petrolatum (Vaselinum album, VA), beeswax (Cera alba, CA), and cetylstearyl alcohol (CSA) was softened in a water bath with continuous stirring. The softened mixture was used to emulsify a mixture of purified water (PW) and polysorbate 60 (P60) heated to the same temperature (50 ± 2 °C) and stirred until cool. The amount of water evaporated was replaced. A solution of methyl para-hydroxybenzoate (MPB) (1 *w*/*V*%) was added to the cooled ointment. Each ointment contained 1.00 g of *C. erythraea* lyophilized product which was pre-dissolved in TP (III.), in CP90 (IV.) and in the mixture of TP-CP90 (V.) and added to the formulations finally. Different ointment formulations are shown in Table 8.

### 4.5. pH Measurement

To determine the pH of the ointments, 1.00 g of each formulation was mixed with 9.00 mL of distilled water in a beaker at 32 °C with continuous stirring to obtain a 10 *w*/*V*% dispersion. After stirring for 5 min, the samples were measured with a pre-calibrated digital portable pH meter (Mettler Toledo, Zurich, Switzerland) [98].

To evaluate the stability of the formulations, pH values were also measured on days 0 and 30. Measuring pH values on days 0. and 30. helps to monitor the stability of the ointment and its effect on the skin, as changes in pH may indicate chemical or microbiological changes in the formulation.

### 4.6. Texture Analysis

Texture analysis of the different formulations was performed using Brookfield CT3 Texture Analyzer (Brookfield, Middleboro, MA, USA). In the analysis, a test probe is penetrated into the sample and the apparatus measures the resistance (nM) exerted by the sample tested. The test probe was a TA10 (12.7 mm D, 35 mm L) cylinder-shaped body, and the test parameters were: trigger force (4 g), peak force (5 mm), velocity (0.5 mm/s). The test was carried out at RT, and the formulation without excipient was used as control. Texture Pro Software version 1.3 (Brookfield Engineering Laboratories, Middleboro, MA, USA) was used to evaluate the results obtained [99].

### 4.7. Viscosity Measurement of Different Formulations

RheolabQC rotational viscometer was used to measure the rheological properties of the different ointment formulations. The viscosity curves of the formulations were evaluated using RheoPlus Rheometer Software (32 V3. 10 21003407-33024) and plotted using GraphPad Prism software (version 10.4; GraphPad Software, San Diego, CA, USA). For the test, 20 g of the mixture was placed in the cup of the cylindrical measuring system and the viscosity was measured at 24 °C. The shear rate was increased from 0.01 s to 100 s, the duration of a measurement was 120 s, and 25 measurement points were obtained for each measurement to determine the viscosity curve [100].

### 4.8. Determination of Diffused API Through Synthetic Membrane for Release Testing

The in vitro drug release from the ointments was assessed using a Franz diffusion cell (Hanson Microette™ Topical and Transdermal Diffusion Cell System, Chatsworth, CA, USA). A synthetic cellulose acetate membrane was employed as a non-biological barrier for release testing to evaluate the diffusion of quercetin from the formulations. The membrane was positioned between the donor and acceptor compartments, and samples were collected at predetermined time intervals (0, 60, 120, 180, and 240 min). The membrane had a diffusion area of 1.767 cm^2^ and a pore diameter of 0.45 µm, while 7 mL of freshly prepared 50% ethyl alcohol served as the acceptor phase. To ensure uniform drug distribution and consistent contact between the donor phase and the membrane, the medium was maintained under continuous magnetic stirring at 300 rpm and a temperature of 32 ± 0.5 °C. Each formulation (0.5 g) was applied to the membrane, corresponding to 2.50 mg of *C. erythraea* in the preparation.

The absorbance of the samples was measured with a UV spectrophotometer (Shimadzu, Tokyo, Japan) at 370 nm to determine the concentration of quercetin. Ethanol (50% *v*/*v*) was used as a solvent and a dilution series was prepared with known concentrations and a calibration curve was determined prior to the measurement.

It should be acknowledged that the cellulose acetate membrane was utilized exclusively as a synthetic barrier for in vitro release assessment; therefore, the obtained results reflect drug release characteristics only and should not be interpreted as indicative of skin permeation [101].

Dissolution profiles were evaluated using multiple approaches. *Flux* was determined with the equation:

(1) $$Flux=\;\frac{Q}{t}$$

The quercetin release rate (*k*) was estimated from the slope of the amount of drug released per unit area (µg/cm^2^) versus the square root of time (min^½^). The diffusion coefficient (*D*) of the drug was determined from the drug concentration at a given *t* time (*Q*, µg/cm^2^), the initial concentration ($C_{0}^{\prime }$), and the diffusion time (*t*):

(2) $$D=\frac{Q^{2}\;\times \;\pi }{(2{{[C}_{0}^{\prime }])}^{2}\times \;t}$$

The dissolution curves were analyzed by fitting the data to several mathematical models, including zero-order, first-order, Korsmeyer–Peppas, Higuchi, and Weibull models, using a graphical approach. These metrics provided a comprehensive comparison of the dissolution and drug release characteristics [102].

### 4.9. In Vitro Antioxidant Activity Tests

#### 4.9.1. DPPH (2,2-Diphenyl-1-picrylhydrazyl) Assay

To assess the antioxidant capacity of the plant, the *DPPH* method was utilized. To prepare the *DPPH* solution, 0.025 g of *DPPH* powder was dissolved in 100 mL of 96% ethanol, resulting in a final concentration of 0.06 mM. The solution was stirred gently to ensure complete dissolution and stored in a dark container to protect it from light. This assay involved the combination of a defined volume of the plant extract with a *DPPH* radical solution (0.06 mM), followed by incubation in the dark at RT. During this process, antioxidants present in the extract facilitate the transfer of hydrogen to the *DPPH* radicals, resulting in a color change from dark violet to light yellow. It can readily undergo reduction by an antioxidant (*AH*) which can be demonstrated by the following reaction [103].

(3) DPPH+AH→DPPH−H+A

The degree of this color change was quantitatively evaluated through absorbance measurements at 517 nm with spectrophotometer (Thermo-Fisher, Waltham, MA, USA). This approach provides a reliable assessment of the plant’s free radical scavenging ability, underscoring its potential antioxidant properties. The antioxidant activity percentage (*AA*%) was determined using the following equation [104].

(4) $$AA\%=100-\frac{\left({Abs}_{sample}-{Abs}_{blank}\right)\times 100}{{Abs}_{control}}$$

#### 4.9.2. FRAP Assay (Ferric Reducing Antioxidant Potential)

The FRAP assay is a commonly used method to measure the antioxidant capacity of a sample based on its ability to reduce ferric ions (Fe^3+^) to ferrous ions (Fe^2+^). In this method, a ferric tripyridyltriazine (Fe^3+^-TPTZ) complex is reduced to a blue-colored ferrous form (Fe^2+^-TPTZ) in the presence of antioxidants. The intensity of the blue color, which is proportional to the antioxidant activity, is measured spectrophotometrically at 590 nm. FRAP assay kit (Sigma-MAK509) was used to test the antioxidant effect and the assay was performed according to the manufacturer’s instructions [105].

#### 4.9.3. Total Polyphenol Content (TPC)

The total phenol content of the different formulations was determined using the Folin–Ciocalteu method. First, 15 µL of test solvent was pipetted into a 96-well plate, and Folin–Ciocalteu reagent (0.2 M) and Na_2_CO_3_ solution (6 *w*/*v*%) and distilled water were added in a 1:1 ratio. The sample was incubated for 30 min in darkness and the absorbance was measured at 760 nm using a spectrophotometer. The total phenol content was calculated from the gallic acid calibration curve and expressed as gallic acid equivalent (GAE) in milligrams per milliliter of sample [106].

### 4.10. Cell Culturing

Immortalized human keratinocyte cells were grown to perform in vitro assays. The cell line was regularly passaged; HaCaT cells were cultured in DMEM supplemented with 3.7 g/L NaHCO_3_, 10 *v*/*v*% FBS, 1 *v*/*v*% non-essential amino acids solution, 1 *v*/*v*% L-glutamine, 100 IU/mL penicillin, and 1001 IU/mL streptomycin. The cell line was maintained in a 75 cm^3^ flask at 37 °C and 5% CO_2_. For in vitro assays, cells were applied between 15 and 25 passages.

### 4.11. MTT Cytotoxicity Assay

The MTT test is a method that can be used to measure cell viability and proliferation. MTT (3-[4,5-dimethylthiazol-2-yl]-2,5-diphenyltetrazolium bromide) is a tetrazolium salt that is converted to bluish-purple formazan crystals by mitochondrial enzymes in metabolically active cells. The amount of transformation is proportional to the viability of the cells. Human keratinocyte cell line (HaCaT) was used to measure viability, and cells were previously seeded in 96-well plates at 10^4^/well density. After reaching full confluency, cells were incubated for 3 h at 37 °C with 5% CO_2_ with test solutions. After the removal of the samples, the cells were washed with PBS and MTT dye (5 mg/mL) was added to each well and incubated for 3 h at 37 °C, in a light-free environment. The dye was removed and the formazan crystals were solubilized in a 95:5 mixture of hydrochloric acid–isopropanol. The absorbance of the solutions was determined using a spectrophotometer at a wavelength of 570 nm, using 690 nm as background wavelength [107].

### 4.12. Investigation of IL-1β, Enzyme-Linked Immunosorbent Assay (ELISA) on HaCaT Cells

The investigation of *C. erythraea* effect on inflammation, sandwich ELISA was carried out using human keratinocyte cells (HaCaT). Cells were seeded on 96-well plates in the density of 10^4^ cells/well. When the cells fully grow over the wells’ membrane, the experiment was performed. Culture media was removed, then TNF-α solution (10 ng/mL) was added to the cells and incubated for 24 h. After 24 h the inflammatory media was removed and cells were washed with PBS, then incubated with the samples for 24 h at 37 °C with 5% CO_2_. Samples were made of lyophilized *C. erythraea* dissolved in PBS, then filtrated (0.2 μm). Supernatant containing the samples was collected, and human IL-1β kit (Sigma-RAB0273) was used to evaluate the anti-inflammatory effect according to the manufacturer’s instructions.

### 4.13. Experimental Animals

The experiments are carried out on young adult albino rats (*Rattus norvegicus*). The rats are weighed on a special scale to ensure accurate dosing. The average weight of the animals was 350 g ± 32 before the beginning of the experiment. The animals are acclimatized for 1 week under laboratory conditions prior to the experiment. They were fed a standard diet and provided with ad libitum water. If any animal showed signs of illness or severe distress (dilated pupils, increased pulse and respiration rate, urinary and fecal excretion, sudden vocalization, restlessness, aggressiveness, assumption of a suppressed posture), the experiment was terminated by euthanasia (cervical dislocation). The rats were kept and treated in accordance with the “Principles of Laboratory Animal Care” established by the National Society for Medical Research, and the “Guide for the Care and Use of Laboratory Animals” by the National Academy of Sciences, published by the National Institutes of Health.

Throughout their life cycle, the inbred animals concerned are housed in polypropylene cages (Sealsafe Plus GR900) of 904 cm^2^ suitable for small animals. The drinkers are glass or clear plastic bottles with chrome-plated steel or glass teat. Approval number: 10/2022/DEMÁB.

### 4.14. In Vivo Carrageenan-Induced Inflammation Assay

The in vivo anti-inflammatory effect was studied by induction of paw edema in preselected rats. Rats were anesthetized with ketamine-xylazine (50/10 mg/kg body weight) before treatment and the paws were subsequently injected with 1% Carragenan solution. Before treatment, the paws of the rats were measured, and after measurement, the paws were treated with the formulation that was found to be more effective based on the in vitro results. Paw edema size was measured at different time intervals (0 h, 3 h and 24 h). In the study, samples were compared to untreated control, and as a positive control, methylprednisolone ointment was used (1 mg/g) [108].

### 4.15. Statistical Analyses

All quantitative data were expressed as mean ± standard deviation (SD) and analyzed using GraphPad Prism software (version 6.0; GraphPad Software, San Diego, CA, USA). One-way analysis of variance (ANOVA) followed by Tukey’s post hoc test was employed to assess statistical differences between groups. A *p*-value less than 0.05 was considered statistically significant, with levels of significance indicated as follows: *p* < 0.05 (*), *p* < 0.01 (**), *p* < 0.001 (***), and *p* < 0.0001 (****). All measurements were performed in replicates (*n* = 3 or *n* = 6, as specified), ensuring reproducibility and reliability of the results.

## 5. Conclusions

In conclusion, this study provides the first comprehensive phytochemical and pharmacological evaluation of *C. erythraea* collected in Romania, emphasizing its unexplored topical applications. Our results prove that ointments formulated with lyophilized extract of *C. erythraea*, mainly those containing both Transcutol^®^ P and Capryol^®^ 90, exhibit remarkable antioxidant and anti-inflammatory properties, effective skin penetration and strong safety profiles. These results suggest considerable potential in the future for C. erythraea-based formulations in the management of inflammatory skin disorders, offering a natural alternative to conventional therapies with fewer side effects.

## Figures and Tables

**Table 1:** Results of phytochemical analysis. The results of phytochemical analysis summarizing the identification and quantification of bioactive compounds in *C. erythraea* extract using both validated LC-MS/MS analytical methods. Concentrations are expressed as mean ± SD (*n* = 3). <LOQ (below limit of quantification.

No.	Compounds	Concentration (µg/mL)
1	Gentisic acid	<LOQ
2	Caffeic acid	0.479 ± 0.052
3	Chlorogenic acid	1.069 ± 0.064
4	4-*O*-caffeoylquinic acid	0.966 ± 0.057
5	Ferulic acid	0.481 ± 0.043
6	Hyperoside	3.778 ± 0.075
7	Isoquercitrin	0.561 ± 0.067
8	Rutin	<LOQ
9	Quercitrin	2.613 ± 0.156
10	Quercetin	0.527 ± 0.026
11	Luteolin	<LOQ
12	Kaempferol	<LOQ
13	Apigenin	<LOQ
14	Epicatechin	0.026 ± 0.002
15	Syringic acid	0.04 ± 0.001
16	Gallic acid	0.181 ± 0.009
17	Protocatechuic acid	1.126 ± 0.101
18	Epigallocatechin gallate	0.179 ± 0.023

**Table 2:** The particle size, PDI and zeta potential of different formulations. Results are expressed as mean ± S.D. (*n* = 3).

Entry	Particle Size_0_ (nm)	PDI_0_	Particle Size_30_ (nm)	PDI_30_	Zeta Potential (mV)	Zeta Potential_30_ (mV)
**1.**	161.42 ± 2.48	0.241 ± 0.014	185.96 ± 7.96	0.417 ± 0.008	−5.570 ± 0.49	−5.324 ± 0.35
**2.**	188.90 ± 0.70	0.303 ± 0.015	240.90 ± 3.70	0.506 ± 0.032	−4.596 ± 0.26	−3.984 ± 0.28
**3.**	113.56 ± 2.04	0.306 ± 0.033	196.67 ± 3.56	0.421 ± 0.006	−6.497 ± 0.56	−6.287 ± 0.42

**Table 3:** pH Values of Ointment Formulations at Day 0 and Day 30. Data expressed as mean ± SD (*n* = 6).

Entry	pH_0_	pH_30_
I.	5.92 ± 0.05	5.87 ± 0.04
II.	5.78 ± 0.04	5.72 ± 0.03
III.	5.47 ± 0.04	5.46 ± 0.06
IV.	5.12 ± 0.03	5.13 ± 0.03
V.	5.39 ± 0.04	5.41 ± 0.04

**Table 4:** Quercetin release rate and the diffusion coefficient values related to the formulations II–V. Each data point represents the mean ± S.D., *n* = 6.

Entry	Release Rate k·10^2^ (µg/cm^2^·min^1/2^) ± SD	Diffusion Coefficient D·10^5^ (cm^2^/min) ± SD
II.	0.41 ± 0.04	7.99 ± 2.13
III.	0.78 ± 0.04	27.05 ± 3.27
IV.	0.81 ± 0.07	23.93 ± 2.19
V.	0.86 ± 0.06	35.12 ± 3.39

**Table 5:** Goodness-of-Fit (R^2^) values for various kinetic models applied to Quercetin release profiles from ointment formulations.

Entry	Zero-Order R^2^	First-Order R^2^	Higuchi R^2^	Korsmeyer–Peppas R^2^	Weibull R^2^
**II.**	0.782	—	0.945	0.973	—
**III.**	0.679	—	0.975	0.986	—
**IV.**	0.797	—	0.929	0.965	—
**V.**	0.536	—	0.978	0.980	—

**Table 6:** Total phenolic content measurement. Results are expressed as milligrams of gallic acid equivalents per milliliter (mg GAE/mL) ± S.D.

Entry	TPC (mg GAE/mL)
II.	59.07 ± 3.43
III.	68.43 ± 1.84
IV.	66.84 ± 0.57
V.	69.71 ± 0.83

**Table 7:** Different compositions of lyophilized *C. erythraea* formulations.

Entry	Lyophilized *C. erythraea* (g)	Transcutol^®^ P (g)	Capryol^®^ 90 (g)
1.	1.00	5.00	-
2.	1.00	-	2.00
3.	1.00	5.00	2.00

**Table 8:** Different formulations containing *C. erythraea* lyophilized product.

Composition	I.(g)	II. (g)	III. (g)	IV. (g)	V. (g)
**White Vaseline**	40.00	40.00	40.00	40.00	40.00
**Cetylstearyl alcohol**	8.00	8.00	8.00	8.00	8.00
**Beeswax**	2.00	2.00	2.00	2.00	2.00
**Polysorbate 60**	2.00	2.00	2.00	2.00	2.00
**Methylparahydroxybenzoate solution (1 *w*/*V*)**	1.00	1.00	1.00	1.00	1.00
**Distilled water**	up to 100.00	up to 100.00	up to 100.00	up to 100.00	up to 100.00
**Transcutol^®^ P**	-	-	5.00	-	5.00
**Capryol^®^ 90**	-	-	-	2.00	2.00
**Lyophilized *C. erythraea***	-	1.00	1.00	1.00	1.00

## Data Availability

The data that support the findings of this study are available from the corresponding author (feher.palma@pharm.unideb.hu) with the permission of the head of the department, upon reasonable request.

## References

[1] Wang H., Chen Y., Wang L., Liu Q., Yang S., Wang C. (2023). Advancing Herbal Medicine: Enhancing Product Quality and Safety through Robust Quality Control Practices. Front. Pharmacol..

[2] Ekor M. (2014). The Growing Use of Herbal Medicines: Issues Relating to Adverse Reactions and Challenges in Monitoring Safety. Front. Pharmacol..

[3] Buhse L., Kolinski R., Westenberger B., Wokovich A., Spencer J., Chen C.W., Turujman S., Gautam-Basak M., Kang G.J., Kibbe A. (2005). Topical Drug Classification. Int. J. Pharm..

[4] Guedes L., Reis P.B.P.S., Machuqueiro M., Ressaissi A., Pacheco R., Serralheiro M.L. (2019). Bioactivities of Centaurium Erythraea (Gentianaceae) Decoctions: Antioxidant Activity, Enzyme Inhibition and Docking Studies. Molecules.

[5] Šiler B., Mišić D. (2016). Biologically Active Compounds from the Genus Centaurium s.l. (Gentianaceae). Stud. Nat. Prod. Chem..

[6] Kültür Ş. (2007). Medicinal Plants Used in Kırklareli Province (Turkey). J. Ethnopharmacol..

[7] Tahraoui A., Israili Z.H., Lyoussi B. (2010). Acute and Sub-Chronic Toxicity of a Lyophilised Aqueous Extract of Centaurium Erythraea in Rodents. J. Ethnopharmacol..

[8] Đorđević M., Grdović N., Mihailović M., Arambašić Jovanović J., Uskoković A., Rajić J., Sinadinović M., Tolić A., Mišić D., Šiler B. (2019). Centaurium Erythraea Extract Improves Survival and Functionality of Pancreatic Beta-Cells in Diabetes through Multiple Routes of Action. J. Ethnopharmacol..

[9] Petrović A., Madić V., Stojanović G., Zlatanović I., Zlatković B., Vasiljević P., Đorđević L. (2024). Antidiabetic Effects of Polyherbal Mixture Made of Centaurium Erythraea, Cichorium Intybus and Potentilla Erecta. J. Ethnopharmacol..

[10] El Menyiy N., Guaouguaou F.-E., El Baaboua A., El Omari N., Taha D., Salhi N., Shariati M.A., Aanniz T., Benali T., Zengin G. (2021). Phytochemical Properties, Biological Activities and Medicinal Use of Centaurium Erythraea Rafn. J. Ethnopharmacol..

[11] Berkan T., Üstünes L., Lermioglu F., Özer A. (1991). Antiinflammatory, Analgesic, and Antipyretic Effects of an Aqueous Extract of *Erythraea Centaurium*. Planta Med..

[12] Aberham A., Pieri V., Croom E.M., Ellmerer E., Stuppner H. (2011). Analysis of Iridoids, Secoiridoids and Xanthones in Centaurium Erythraea, Frasera Caroliniensis and Gentiana Lutea Using LC-MS and RP-HPLC. J. Pharm. Biomed. Anal..

[13] Kumarasamy Y., Nahar L., Cox P.J., Jaspars M., Sarker S.D. (2003). Bioactivity of Secoiridoid Glycosides from Centaurium Erythraea. Phytomedicine.

[14] Oresajo C., Pillai S., Manco M., Yatskayer M., McDaniel D. (2012). Antioxidants and the Skin: Understanding Formulation and Efficacy. Dermatol. Ther..

[15] Chen J., Liu Y., Zhao Z., Qiu J. (2021). Oxidative Stress in the Skin: Impact and Related Protection. Int. J. Cosmet. Sci..

[16] Valentão P., Fernandes E., Carvalho F., Andrade P.B., Seabra R.M., Bastos M.L. (2001). Antioxidant Activity of *Centaurium Erythraea* Infusion Evidenced by Its Superoxide Radical Scavenging and Xanthine Oxidase Inhibitory Activity. J. Agric. Food Chem..

[17] Serhan C.N. (2017). Treating Inflammation and Infection in the 21st Century: New Hints from Decoding Resolution Mediators and Mechanisms. FASEB J..

[18] Kunnumakkara A.B., Sailo B.L., Banik K., Harsha C., Prasad S., Gupta S.C., Bharti A.C., Aggarwal B.B. (2018). Chronic Diseases, Inflammation, and Spices: How Are They Linked?. J. Transl. Med..

[19] Van Linthout S., Tschöpe C. (2017). Inflammation – Cause or Consequence of Heart Failure or Both?. Curr. Heart Fail. Rep..

[20] Chen L., Deng H., Cui H., Fang J., Zuo Z., Deng J., Li Y., Wang X., Zhao L. (2018). Inflammatory Responses and Inflammation-Associated Diseases in Organs. Oncotarget.

[21] Slavich G.M., Irwin M.R. (2014). From Stress to Inflammation and Major Depressive Disorder: A Social Signal Transduction Theory of Depression. Psychol. Bull..

[22] Pelaia G., Vatrella A., Busceti M.T., Gallelli L., Calabrese C., Terracciano R., Maselli R. (2015). Cellular Mechanisms Underlying Eosinophilic and Neutrophilic Airway Inflammation in Asthma. Mediators Inflamm..

[23] Esser N., Legrand-Poels S., Piette J., Scheen A.J., Paquot N. (2014). Inflammation as a Link between Obesity, Metabolic Syndrome and Type 2 Diabetes. Diabetes Res. Clin. Pract..

[24] Griffiths C.E.M., van de Kerkhof P., Czarnecka-Operacz M. (2017). Psoriasis and Atopic Dermatitis. Dermatol. Ther..

[25] Barnes P.J. (2014). Glucocorticoids. Chem. Immunol. Allergy.

[26] Sohail R., Mathew M., Patel K.K., Reddy S.A., Haider Z., Naria M., Habib A., Abdin Z.U., Razzaq Chaudhry W., Akbar A. (2023). Effects of Non-Steroidal Anti-Inflammatory Drugs (NSAIDs) and Gastroprotective NSAIDs on the Gastrointestinal Tract: A Narrative Review. Cureus.

[27] De Sá Coutinho D., Pacheco M.T., Frozza R.L., Bernardi A. (2018). Anti-Inflammatory Effects of Resveratrol: Mechanistic Insights. Int. J. Mol. Sci..

[28] Yatoo M.I., Gopalakrishnan A., Saxena A., Parray O.R., Tufani N.A., Chakraborty S., Tiwari R., Dhama K., Iqbal H.M.N. (2018). Anti-Inflammatory Drugs and Herbs with Special Emphasis on Herbal Medicines for Countering Inflammatory Diseases and Disorders—A Review. Recent Pat. Inflamm. Allergy Drug Discov..

[29] Ge J., Liu Z., Zhong Z., Wang L., Zhuo X., Li J., Jiang X., Ye X.-Y., Xie T., Bai R. (2022). Natural Terpenoids with Anti-Inflammatory Activities: Potential Leads for Anti-Inflammatory Drug Discovery. Bioorg. Chem..

[30] Patel S.S., Savjani J.K. (2015). Systematic Review of Plant Steroids as Potential Antiinflammatory Agents: Current Status and Future Perspectives. J. Phytopharm..

[31] Gonfa Y.H., Tessema F.B., Bachheti A., Rai N., Tadesse M.G., Nasser Singab A., Chaubey K.K., Bachheti R.K. (2023). Anti-Inflammatory Activity of Phytochemicals from Medicinal Plants and Their Nanoparticles: A Review. Curr. Res. Biotechnol..

[32] Censi R., Martena V., Hoti E., Malaj L., Di Martino P. (2012). Permeation and Skin Retention of Quercetin from Microemulsions Containing Transcutol ^®^ P. Drug Dev. Ind. Pharm..

[33] Hernandez C., Jain P., Sharma H., Lam S., Sonti S. (2020). Investigating the Effect of Transcutol on the Physical Properties of an O/W Cream. J. Dispers. Sci. Technol..

[34] Shakeel F., Haq N., Alanazi F.K., Alsarra I.A. (2013). Impact of Various Nonionic Surfactants on Self-Nanoemulsification Efficiency of Two Grades of Capryol (Capryol-90 and Capryol-PGMC). J. Mol. Liq..

[35] Musakhanian J., Osborne D.W., Rodier J.-D. (2024). Skin Penetration and Permeation Properties of Transcutol® in Complex Formulations. AAPS PharmSciTech.

[36] Birngruber T., Vought K., Schwingenschuh S., Reisenegger P., Maibach H., Lissin D. (2023). Topical Delivery Systems Effectively Transport Analgesics to Areas of Localized Pain via Direct Diffusion. Pharmaceutics.

[37] INAKI T. (2001). Ointments for Antiinflammatory Drugs. Gels Handbook.

[38] Sandru D., Niculescu V., Lengyel E., Tița O. (2016). Identification and Quantification of Total Polyphenols in Plants with Bioactive Potentially. Int. J. Pharmacol. Phytochem. Ethnomed..

[39] Brudzińska-Kosior A., Kosior G., Sporek M., Ziembik Z., Zinicovscaia I., Frontasyeva M., Dołhańczuk-Śródka A. (2023). Nuclear Analytical Techniques Used to Study the Trace Element Content of Centaurium Erythraea Rafn, a Medicinal Plant Species from Sites with Different Pollution Loads in Lower Silesia (SW Poland). PLoS ONE.

[40] Chda A., El Kabbaoui M., Fresco P., Silva D., Gonçalves J., Oliveira A.P., Andrade P.B., Valentão P., Tazi A., El Abida K. (2020). Centaurium Erythraea Extracts Exert Vascular Effects through Endothelium- and Fibroblast-Dependent Pathways. Planta Med..

[41] Németh Z., Csóka I., Semnani Jazani R., Sipos B., Haspel H., Kozma G., Kónya Z., Dobó D.G. (2022). Quality by Design-Driven Zeta Potential Optimisation Study of Liposomes with Charge Imparting Membrane Additives. Pharmaceutics.

[42] Yang L., Wen K.-S., Ruan X., Zhao Y.-X., Wei F., Wang Q. (2018). Response of Plant Secondary Metabolites to Environmental Factors. Molecules.

[43] Brudzińska-Kosior A., Kosior G., Samecka-Cymerman A., Kolon K., Mróz L., Kempers A.J. (2012). Metal Contents in Centaurium Erythraea and Its Biometry at Various Levels of Environmental Pollution. Ecotoxicol. Environ. Saf..

[44] Mijajlovic N., Grubisic D., Giba Z., Konjevic R. (2005). The Effect of Plant Growth Regulators on Centaury (Centaurium Erythraea Rafn) Seeds Germination. Arch. Biol. Sci..

[45] Reiné R., Chocarro C., Fillat F. (2006). Spatial Patterns in Seed Bank and Vegetation of Semi-Natural Mountain Meadows. Plant Ecol..

[46] Mihaylova D., Vrancheva R., Popova A. (2019). Phytochemical Profile and in Vitro Antioxidant Activity of Centaurium Erythraea Rafn. Bulg. Chem. Commun..

[47] ElNaker N.A., Daou M., Ochsenkühn M.A., Amin S.A., Yousef A.F., Yousef L.F. (2021). A Metabolomics Approach to Evaluate the Effect of Lyophilization versus Oven Drying on the Chemical Composition of Plant Extracts. Sci. Rep..

[48] Nwadibia J.A., Fasogbon I.V., Musyoka A.M., Ekpono E.U., Ibiam U.A., Orji O.U., Eze E.D., Onaadepo O., Agu P.C., Aja P.M. (2024). Protective Effect of Ficus Capensis Lyophilized Extract against Carboplatin-Induced Liver Injury via Inhibition of Oxidative Stress and Inflammation in Rats. Toxicol. Rep..

[49] Sibri J.F., Akakpo-Akue J., Okou O.C., Kple T.K.M. (2023). Evaluation of the Antioxidant Activity of Aqueous and Hydro-Ethanolic Extracts of Ficus Capensis, Newbouldia Laevis and Carpolobia Lutea. Asian J. Res. Biochem..

[50] Naveed M., Hejazi V., Abbas M., Kamboh A.A., Khan G.J., Shumzaid M., Ahmad F., Babazadeh D., FangFang X., Modarresi-Ghazani F. (2018). Chlorogenic Acid (CGA): A Pharmacological Review and Call for Further Research. Biomed. Pharmacother..

[51] Zduńska K., Dana A., Kolodziejczak A., Rotsztejn H. (2018). Antioxidant Properties of Ferulic Acid and Its Possible Application. Skin Pharmacol. Physiol..

[52] Spagnol C.M., Di Filippo L.D., Isaac V.L.B., Correa M.A., Salgado H.R.N. (2017). Caffeic Acid in Dermatological Formulations: In Vitro Release Profile and Skin Absorption. Comb. Chem. High Throughput Screen..

[53] Song H.S., Park T.W., Sohn U.D., Shin Y.K., Choi B.C., Kim C.J., Sim S.S. (2008). The Effect of Caffeic Acid on Wound Healing in Skin-Incised Mice. Korean J. Physiol. Pharmacol..

[54] Shin S., Cho S.H., Park D., Jung E. (2020). Anti-skin Aging Properties of Protocatechuic Acid in Vitro and in Vivo. J. Cosmet. Dermatol..

[55] Hadidi M., Liñán-Atero R., Tarahi M., Christodoulou M.C., Aghababaei F. (2024). The Potential Health Benefits of Gallic Acid: Therapeutic and Food Applications. Antioxidants.

[56] Yang D., Moh S., Son D., You S., Kinyua A., Ko C., Song M., Yeo J., Choi Y.-H., Kim K. (2016). Gallic Acid Promotes Wound Healing in Normal and Hyperglucidic Conditions. Molecules.

[57] Okselni T., Septama A.W., Juliadmi D., Dewi R.T., Angelina M., Yuliani T., Saragih G.S., Saputri A. (2025). Quercetin as a Therapeutic Agent for Skin Problems: A Systematic Review and Meta-Analysis on Antioxidant Effects, Oxidative Stress, Inflammation, Wound Healing, Hyperpigmentation, Aging, and Skin Cancer. Naunyn. Schmiedebergs. Arch. Pharmacol..

[58] Beken B., Serttas R., Yazicioglu M., Turkekul K., Erdogan S. (2020). Quercetin Improves Inflammation, Oxidative Stress, and Impaired Wound Healing in Atopic Dermatitis Model of Human Keratinocytes. Pediatr. Allergy. Immunol. Pulmonol..

[59] Mapoung S., Umsumarng S., Semmarath W., Arjsri P., Srisawad K., Thippraphan P., Yodkeeree S., Dejkriengkraikul P. (2021). Photoprotective Effects of a Hyperoside-Enriched Fraction Prepared from Houttuynia Cordata Thunb. on Ultraviolet B-Induced Skin Aging in Human Fibroblasts through the MAPK Signaling Pathway. Plants.

[60] Moukova A., Malina L., Kolarova H., Bajgar R. (2023). Hyperoside as a UV Photoprotective or Photostimulating Compound—Evaluation of the Effect of UV Radiation with Selected UV-Absorbing Organic Compounds on Skin Cells. Int. J. Mol. Sci..

[61] Li Y., Ma Y., Yao Y., Ru G., Lan C., Li L., Huang T. (2024). Protective Effect of Isoquercitrin on UVB-induced injury in HaCaT cells and mice skin through anti-inflammatory, antioxidant, and regulation of MAPK and JAK2-STAT3 Pathways. Photochem. Photobiol..

[62] Lee E., Park H., Kim H., Jung H., Kang I., Cho Y. (2021). Isolated Isoquercitrin from Green Ball Apple Peel Inhibits Photoaging in CCD-986Sk Fibroblasts Cells via Modulation of the MMPs Signaling. J. Cosmet. Dermatol..

[63] Murai T., Matsuda S. (2023). The Chemopreventive Effects of Chlorogenic Acids, Phenolic Compounds in Coffee, against Inflammation, Cancer, and Neurological Diseases. Molecules.

[64] LI L., ZHANG X.-H., LIU G.-R., LIU C., DONG Y.-M. (2016). Isoquercitrin Suppresses the Expression of Histamine and Pro-Inflammatory Cytokines by Inhibiting the Activation of MAP Kinases and NF-ΚB in Human KU812 Cells. Chin. J. Nat. Med..

[65] Kuppan G., Balasubramanyam J., Monickaraj F., Srinivasan G., Mohan V., Balasubramanyam M. (2010). Transcriptional Regulation of Cytokines and Oxidative Stress by Gallic Acid in Human THP-1 Monocytes. Cytokine.

[66] Stefkov G., Miova B., Dinevska-Kjovkarovska S., Stanoeva J.P., Stefova M., Petrusevska G., Kulevanova S. (2014). Chemical Characterization of Centaurium Erythrea L. and Its Effects on Carbohydrate and Lipid Metabolism in Experimental Diabetes. J. Ethnopharmacol..

[67] Mitsutake H., Ribeiro L.N.M., Rodrigues da Silva G.H., Castro S.R., de Paula E., Poppi R.J., Breitkreitz M.C. (2019). Evaluation of Miscibility and Polymorphism of Synthetic and Natural Lipids for Nanostructured Lipid Carrier (NLC) Formulations by Raman Mapping and Multivariate Curve Resolution (MCR). Eur. J. Pharm. Sci..

[68] Braun-Falco O., Korting H.C. (1986). Normal PH Value of Human Skin. Hautarzt.

[69] Jia Y., Gan Y., He C., Chen Z., Zhou C. (2018). The Mechanism of Skin Lipids Influencing Skin Status. J. Dermatol. Sci..

[70] Danso M., Boiten W., van Drongelen V., Gmelig Meijling K., Gooris G., El Ghalbzouri A., Absalah S., Vreeken R., Kezic S., van Smeden J. (2017). Altered Expression of Epidermal Lipid Bio-Synthesis Enzymes in Atopic Dermatitis Skin Is Accompanied by Changes in Stratum Corneum Lipid Composition. J. Dermatol. Sci..

[71] Seweryn A. (2018). Interactions between Surfactants and the Skin – Theory and Practice. Adv. Colloid Interface Sci..

[72] Wagner H. (2003). PH Profiles in Human Skin: Influence of Two in Vitro Test Systems for Drug Delivery Testing. Eur. J. Pharm. Biopharm..

[73] Bachhav Y.G., Patravale V.B. (2009). SMEDDS of Glyburide: Formulation, In Vitro Evaluation, and Stability Studies. AAPS PharmSciTech.

[74] Osborne D.W., Musakhanian J. (2018). Skin Penetration and Permeation Properties of Transcutol®—Neat or Diluted Mixtures. AAPS PharmSciTech.

[75] Trommer H., Neubert R.H.H. (2006). Overcoming the Stratum Corneum: The Modulation of Skin Penetration. Skin Pharmacol. Physiol..

[76] Godwin D.A., Kim N.-H., Felton L.A. (2002). Influence of Transcutol® CG on the Skin Accumulation and Transdermal Permeation of Ultraviolet Absorbers. Eur. J. Pharm. Biopharm..

[77] Björklund S., Pham Q.D., Jensen L.B., Knudsen N.Ø., Nielsen L.D., Ekelund K., Ruzgas T., Engblom J., Sparr E. (2016). The Effects of Polar Excipients Transcutol and Dexpanthenol on Molecular Mobility, Permeability, and Electrical Impedance of the Skin Barrier. J. Colloid Interface Sci..

[78] Algahtani M.S., Ahmad M.Z., Ahmad J. (2020). Nanoemulgel for Improved Topical Delivery of Retinyl Palmitate: Formulation Design and Stability Evaluation. Nanomaterials.

[79] Korhonen M., Hellen L., Hirvonen J., Yliruusi J. (2001). Rheological Properties of Creams with Four Different Surfactant Combinations—Effect of Storage Time and Conditions. Int. J. Pharm..

[80] Sarkar R., Mandal N. (2012). Hydroalcoholic Extracts of Indian Medicinal Plants Can Help in Amelioration from Oxidative Stress through Antioxidant Properties. J. Complement. Integr. Med..

[81] Šiler B., Živković S., Banjanac T., Cvetković J., Nestorović Živković J., Ćirić A., Soković M., Mišić D. (2014). Centauries as Underestimated Food Additives: Antioxidant and Antimicrobial Potential. Food Chem..

[82] Cecchi L., Piazzini V., D’Ambrosio M., Luceri C., Rocco F., Innocenti M., Vanti G., Mulinacci N., Bergonzi M.C. (2020). Formulation of a Phenol-Rich Extract from Unripe Olives (Olea Europaea L.) in Microemulsion to Improve Its Solubility and Intestinal Permeability. Molecules.

[83] Valicherla G.R., Dave K.M., Syed A.A., Riyazuddin M., Gupta A.P., Singh A., Wahajuddin, Mitra K., Datta D., Gayen J.R. (2016). Formulation Optimization of Docetaxel Loaded Self-Emulsifying Drug Delivery System to Enhance Bioavailability and Anti-Tumor Activity. Sci. Rep..

[84] Mony R.S. (2023). Studies on Anti-Inflammatory Activity of Herbal Extract Mixture Using Inflammation Induced Human Keratinocytes (HaCaT) Cells. J. Pharmacogn. Phytochem..

[85] López-García J., Lehocký M., Humpolíček P., Sáha P. (2014). HaCaT Keratinocytes Response on Antimicrobial Atelocollagen Substrates: Extent of Cytotoxicity, Cell Viability and Proliferation. J. Funct. Biomater..

[86] Deyrieux A.F., Wilson V.G. (2007). In Vitro Culture Conditions to Study Keratinocyte Differentiation Using the HaCaT Cell Line. Cytotechnology.

[87] Saah S., Wiwattanapatapee R. (2018). Cytotoxic Effect of Surfactants Used in Self-Microemulsifying Drug Delivery Systems (SMEDDS) on Normal and Cancer Gastrointestinal Cell Lines. Lat. Am. J. Pharm..

[88] Schwartz C., Moran T., Saunders S.P., Kaszlikowska A., Floudas A., Bom J., Nunez G., Iwakura Y., O’Neill L., Irvine A.D. (2019). Spontaneous Atopic Dermatitis in Mice with a Defective Skin Barrier Is Independent of ILC2 and Mediated by IL-1β. Allergy.

[89] Vazquez E., Navarro M., Salazar Y., Crespo G., Bruges G., Osorio C., Tortorici V., Vanegas H., López M. (2015). Systemic Changes Following Carrageenan-Induced Paw Inflammation in Rats. Inflamm. Res..

[90] Luger T. (2011). Balancing Efficacy and Safety in the Management of Atopic Dermatitis: The Role of Methylprednisolone Aceponate. J. Eur. Acad. Dermatol. Venereol..

[91] Torrelo A. (2017). Methylprednisolone Aceponate for Atopic Dermatitis. Int. J. Dermatol..

[92] Vlase A.-M., Toiu A., Tomuță I., Vlase L., Muntean D., Casian T., Fizeșan I., Nadăș G.C., Novac C.Ș., Tămaș M. (2022). *Epilobium* Species: From Optimization of the Extraction Process to Evaluation of Biological Properties. Antioxidants.

[93] Gligor O., Clichici S., Moldovan R., Muntean D., Vlase A.-M., Nadăș G.C., Matei I.A., Filip G.A., Vlase L., Crișan G. (2023). The Effect of Extraction Methods on Phytochemicals and Biological Activities of Green Coffee Beans Extracts. Plants.

[94] Solcan M.-B., Fizeșan I., Vlase L., Vlase A.-M., Rusu M.E., Mateș L., Petru A.-E., Creștin I.-V., Tomuțǎ I., Popa D.-S. (2023). Phytochemical Profile and Biological Activities of Extracts Obtained from Young Shoots of Blackcurrant (*Ribes Nigrum* L.), European Blueberry (*Vaccinium Myrtillus* L.), and Mountain Cranberry (*Vaccinium Vitis-Idaea* L.). Horticulturae.

[95] Solcan M.-B., Vlase A.-M., Marc G., Muntean D., Casian T., Nadăș G.C., Novac C., Ștefania, Popa D.-S., Vlase L. (2024). Antimicrobial Effectiveness of Ribes Nigrum L. Leaf Extracts Prepared in Natural Deep Eutectic Solvents (NaDESs). Antibiotics.

[96] Karbab A., Charef N., Jaber A.M., Siedat A.M., Kharchi M., Nini R., Sanabrah A. (2025). Antioxidant, Anti-Inflammatory Effects of Centaurium Erythraea Rafn. Aerial Part Extracts and Identification of Its Bioactive Constituents by LC-MS/MS Analysis. Int. J. Comput. Exp. Sci. Eng..

[97] Gaber D.A., Alsubaiyel A.M., Alabdulrahim A.K., Alharbi H.Z., Aldubaikhy R.M., Alharbi R.S., Albishr W.K., Mohamed H.A. (2023). Nano-Emulsion Based Gel for Topical Delivery of an Anti-Inflammatory Drug: In Vitro and in Vivo Evaluation. Drug Des. Devel. Ther..

[98] Bhagurkar A.M., Angamuthu M., Patil H., Tiwari R.V., Maurya A., Hashemnejad S.M., Kundu S., Murthy S.N., Repka M.A. (2016). Development of an Ointment Formulation Using Hot-Melt Extrusion Technology. AAPS PharmSciTech.

[99] Jurca T., Józsa L., Suciu R., Pallag A., Marian E., Bácskay I., Mureșan M., Stan R.L., Cevei M., Cioară F. (2020). Formulation of Topical Dosage Forms Containing Synthetic and Natural Anti-Inflammatory Agents for the Treatment of Rheumatoid Arthritis. Molecules.

[100] Ivko T., Hrytsenko V., Kienko L., Bobrytska L., Kukhtenko H., Germanyuk T. (2021). Investigation of the Rheological Properties of Ointment Bases as a Justification of the Ointment Composition for Herpes Treatment. Turkish J. Pharm. Sci..

[101] Kocabaş N.Ö., Kahraman E., Güngör S. (2021). Assessment of Membrane Type Effects on in Vitro Performance of Topical Semi-Solid Products. J. Drug Deliv. Sci. Technol..

[102] Zsikó S., Cutcher K., Kovács A., Budai-Szűcs M., Gácsi A., Baki G., Csányi E., Berkó S. (2019). Nanostructured Lipid Carrier Gel for the Dermal Application of Lidocaine: Comparison of Skin Penetration Testing Methods. Pharmaceutics.

[103] Gavra D.I., Endres L., Pető Á., Józsa L., Fehér P., Ujhelyi Z., Pallag A., Marian E., Vicas L.G., Ghitea T.C. (2022). In Vitro and Human Pilot Studies of Different Topical Formulations Containing Rosa Species for the Treatment of Psoriasis. Molecules.

[104] Baliyan S., Mukherjee R., Priyadarshini A., Vibhuti A., Gupta A., Pandey R.P., Chang C.-M. (2022). Determination of Antioxidants by DPPH Radical Scavenging Activity and Quantitative Phytochemical Analysis of Ficus Religiosa. Molecules.

[105] Morar I.I., Pop R.M., Peitzner E., Ranga F., Orăsan M.S., Cecan A.D., Chera E.I., Bonci T.I., Usatiuc L.O., Țicolea M. (2025). Phytochemical Composition and Antioxidant Activity of Manuka Honey and Ohia Lehua Honey. Nutrients.

[106] Michiu D., Socaciu M.-I., Fogarasi M., Jimborean A.M., Ranga F., Mureşan V., Semeniuc C.A. (2022). Implementation of an Analytical Method for Spectrophotometric Evaluation of Total Phenolic Content in Essential Oils. Molecules.

[107] Papp B., Le Borgne M., Perret F., Marminon C., Józsa L., Pető Á., Kósa D., Nagy L., Kéki S., Ujhelyi Z. (2023). Formulation and Investigation of CK2 Inhibitor-Loaded Alginate Microbeads with Different Excipients. Pharmaceutics.

[108] Morris C.J. Carrageenan-Induced Paw Edema in the Rat and Mouse. Inflammation Protocols.

